# Effects of the Timing of Herbivory on Plant Defense Induction and Insect Performance in Ribwort Plantain (*Plantago lanceolata* L.) Depend on Plant Mycorrhizal Status

**DOI:** 10.1007/s10886-015-0644-0

**Published:** 2015-11-09

**Authors:** Minggang Wang, T. Martijn Bezemer, Wim H. van der Putten, Arjen Biere

**Affiliations:** Department of Terrestrial Ecology, Netherlands Institute of Ecology (NIOO-KNAW), Droevendaalsesteeg 10, 6708 PB Wageningen, The Netherlands; Laboratory of Nematology, Wageningen University, P.O. Box 8132, 6700 ES Wageningen, The Netherlands

**Keywords:** Arbuscular mycorrhizal fungi, *Plantago lanceolata*, Induced defense, Timing, Above-belowground interactions, Iridoid glycosides

## Abstract

Plants often are exposed to antagonistic and symbiotic organisms both aboveground and belowground. Interactions between above- and belowground organisms may occur either simultaneously or sequentially, and jointly can determine plant responses to future enemies. However, little is known about time-dependency of such aboveground-belowground interactions. We examined how the timing of a 24 h period of aboveground herbivory by *Spodoptera exigua* (1–8 d prior to later arriving conspecifics) influenced the response of *Plantago lanceolata* and the performance of later arriving conspecifics. We also examined whether these induced responses were modulated by the arbuscular mycorrhizal fungus (AMF) *Funneliformis mosseae.* The amount of leaf area consumed by later arriving herbivores decreased with time after induction by early herbivores. Mycorrhizal infection reduced the relative growth rate (RGR) of later arriving herbivores, associated with a reduction in efficiency of conversion of ingested food rather than a reduction in relative consumption rates. In non-mycorrhizal plants, leaf concentrations of the defense compound catalpol showed a linear two-fold increase during the eight days following early herbivory. By contrast, mycorrhizal plants already had elevated levels of leaf catalpol prior to their exposure to early herbivory and did not show any further increase following herbivory. These results indicate that AMF resulted in a systemic induction, rather than priming of these defenses. AMF infection significantly reduced shoot biomass of *Plantago lanceolata*. We conclude that plant responses to future herbivores are not only influenced by exposure to prior aboveground and belowground organisms, but also by when these prior organisms arrive and interact.

## Introduction

Virtually all plants in natural communities experience damage from above- and belowground organisms. In response to damage, primary and secondary metabolites and physical resistance traits often change (Karban and Baldwin 1997; Underwood 2012). Hence, via these herbivore-induced changes, the susceptibility of a plant to later arriving herbivores that feed on the plant can be altered (Kaplan and Denno 2007; Thaler et al. 2002). The impact of herbivory on later arriving herbivores depends on the specific combination of plants and attackers, and both induced resistance and induced susceptibility have been reported to occur as a response to herbivory (Koricheva et al. 2009).

In recent years, the significance of the timing of herbivory in regulating plant-herbivore interactions has been increasingly recognized (Blossey and Hunt-Joshi 2003; Erb et al. 2011; Johnson et al. 2012; Nykänen and Koricheva 2004; Sullivan and Howe 2009). The time lag between damage and the onset of defense, as well as between cessation of damage and the relaxation of defense, are crucial in determining the establishment or feeding of later arriving herbivores (Karban 2011). Both lags may depend on the plant species maintaining the induced defense, but also on the timing of herbivory in relation to plant ontogeny (Boege and Marquis 2005; Gomez et al. 2010; Wang et al. 2014; Young et al. 2003). Generally, younger plants are easier to be induced, and their induced defenses show more plastic responses to other biotic or abiotic factors, while older plants that take more time to induce defenses typically maintain these induced defenses for a longer time (Fuchs and Bowers 2004).

Plant-induced responses to herbivory are not restricted to locally damaged organs, but also can be systemically expressed in undamaged tissues (Bezemer and Van Dam 2005; Van Dam et al. 2004). Several studies have shown that plant responses to herbivores can be altered by the plant’s interaction with belowground microbial plant symbionts such as mycorrhizal fungi and plant growth promoting rhizobacteria (Pangesti et al. 2013; Pineda et al. 2010; Pozo and Azcón-Aguilar 2007; Van Oosten et al. 2008; Zamioudis and Pieterse 2012). Mycorrhizal fungi are root-associated organisms that can influence a plant’s response to herbivory via a diversity of mechanisms. The nutritional status, level of secondary metabolites, and tolerance to abiotic and biotic stress of a plant can all be altered by interactions between the plant and mycorrhizal fungi (Smith and Read 2008). This subsequently can alter the plant responses to its herbivores. These mycorrhizae-induced changes in the plant can be either beneficial or detrimental for herbivores that feed on the plant, and the strength and direction of these effects may depend on the feeding mode or specialization of the herbivore (Bennett et al. 2006; Borowicz 2013; Koricheva et al. 2009). A meta-analysis of 34 studies showed that arbuscular mycorrhizal fungi (AMF) predominantly have negative effects on the performance of generalist chewing herbivores, but that they can enhance the performance of specialist chewing herbivores (Koricheva et al. 2009). Plant secondary compounds often are toxic for generalists, but can be used as feeding stimulants by specialist chewers (Agrawal 2003; Giamoustaris and Mithen 1995). Hence, changes in the production of these chemicals have been proposed as a mechanism by which AMF modulate interactions between a plant and its herbivores (Bennett et al. 2009; De Deyn et al. 2009). Arbuscular mycorrhizal fungi can modulate shoot levels of secondary metabolites in two ways. First, AMF can simply induce defense metabolites in shoots. Second, mycorrhizal infection can modulate the plant’s ability to respond to herbivores, causing a stronger or faster increase in the concentration of defense chemicals in the shoots in response to herbivory (Jung et al. 2012; Song et al. 2013), a phenomenon known as defense priming (Conrath et al. 2006; Pozo and Azcón-Aguilar 2007).

So far, we are not aware of any studies that have explored how AMF interfere with the timing of induction following herbivory and its consequences for the performance of later arriving herbivores. In the current study, we tested whether and how mycorrhization influences the time course of induction of plant defense compounds, and how it affects the performance of later arriving herbivores. To examine how mycorrhization interacts with timing of herbivory we exposed mycorrhizal and non-mycorrhizal *Plantago lanceolata* plants to controlled levels of herbivory at different times prior to introducing response herbivores.

*Plantago lanceolata* L. (Plantaginaceae) (ribwort plantain) is a short-lived perennial forb with a worldwide distribution. It can associate with a multitude of species of AM fungi in the field (Johnson et al. 2004), and it is employed frequently as a model system in studies of plant-mycorrhiza interactions (e.g., Bennett et al. 2009). *Plantago lanceolata* produces several classes of secondary metabolites that can be induced by herbivory (Sutter and Müller 2011). An important class is the iridoid glycosides (IGs), whose levels (mainly aucubin and catalpol) can constitute up to more than 10 % of leaf dry weight (Bowers et al. 1992). These compounds are toxic or deterrent to non-adapted generalist herbivores (Bowers and Puttick 1988; Bowers and Stamp 1992; Darrow and Bowers 1999; Harvey et al. 2005; Reudler et al. 2011) but serve as feeding or oviposition cues for specialists (e.g., Nieminen et al. 2003; Reudler et al. 2008). We chose to focus on these compounds since their tissue levels in *P. lanceolata* are known to be influenced by both herbivory (e.g., Fuchs and Bowers 2004) and by colonization with AM fungi (e.g., Bennett et al. 2009). The induction of IGs by herbivores depends e.g., on the ontogeny of the plant (Quintero and Bowers 2011, 2012) and on the time lag between induction and response (Fuchs and Bowers 2004). Strength and direction of induction of IGs by mycorrhizae in *P. lanceolata* strongly depends on AMF species (Bennett et al. 2009) and varies among studies (e.g., Fontana et al. 2009; Gange and West 1994; Schweiger et al. 2014). Mycorrhization of *P. lanceolata* has been shown to suppress plant induced responses to aboveground herbivory and to alter the proportion of catalpol in the total IG level following herbivory (Bennett et al. 2009, 2013).

As AMF species we used *Funneliformis mosseae* (T.H. Nicolson & Gerd.) C. Walker & A. Schüßler (Glomeraceae) (formerly *Glomus mosseae*)*. Funneliformis mosseae* forms a symbiotic relationship with many plant species including *P. lanceolata* (Karasawa et al. 2012; Orlowska et al. 2012). It has been used in previous studies on plant-mediated AMF-herbivore interactions in other systems, both at the phenotypic (Borowicz 2013) and molecular level (Fernandez et al. 2014; Song et al. 2013). As representative of an aboveground generalist chewing herbivorous insect, we used the southern beet armyworm, *Spodoptera exigua* (Hübner) (Lepidoptera: Noctuidae), that attacks a wide range of plant species (Greenberg et al. 2001). It originates from Southeast Asia, but nowadays has a global distribution. The larvae go through five instars during development and can produce several generations per year.

We tested three hypotheses: i) Plants need time to activate induced defense, and the induced defense decays over time. Hence, early or late timing of prior damage relative to the arrival of later herbivores will result in lower levels of induced defense compounds (IGs) and in a better performance of these herbivores than when prior damage occurs at intermediate time point. ii) AM fungi will prime the plant for a quicker or stronger response to herbivory. Therefore, we expect that AMF colonization will either strengthen the induced plant response (IGs level) to later arriving herbivores, or result in a more rapid response, shifting the onset of the response to an earlier time point. iii) AMF colonization will increase shoot biomass and reduce negative effects of previous herbivory on shoot biomass.

## Methods and Materials

### Plants, Herbivores and AMF

Seeds of *P. lanceolata* were obtained from a full-sib cross between two parents originating from a hayfield and a pasture in the Netherlands, respectively. Seeds were surface sterilized using 1 % sodium hypochlorite (sterilized for 1 min followed by 4 times 5 min rinsing with demineralized water), sown on glass beads, and placed in an incubator (16/8 h L/D and 25/20 °C day/night) until seedling emergence.

Eggs of *S. exigua* were obtained from the Laboratory of Entomology, Wageningen University, The Netherlands. After hatching, larvae were reared on artificial diet (Biere et al. 2004) in a growth chamber at 22 °C, 16:8 h L/D photoperiod and at 70 % RH.

*Funneliformis mosseae* inoculum was purchased from Symbiom Ltd. (Lanskroun, Czech Republic) (Strain BEG 198).

### Experimental Set-up

Soil was collected from a restoration grassland (De Mossel, Ede, The Netherlands) where *P. lanceolata* is abundant. In the laboratory, the soil was sieved through a 0.5 cm mesh, homogenized and gamma-sterilized (>25 KGray). The sandy-loam mineral soil was mixed with sterilized sorbix (Damolin, Fur, Denmark) and sand in a 1:2:2 (soil : sand : sorbix, vv^−1^) proportion to promote drainage. A total of 206 pots (9 × 9 × 10 cm) were filled with 600 g of soil-sand-sorbix mixture. The pots were watered with 50 ml of a soil microbial wash extracted from 25 kg fresh soil suspended in 25 L tap water and filtered through 75, 45, and 20 μm filters to obtain a microbial wash that excluded AMF propagules. The microbial wash was added to establish a background microbial community in the sterile substrate mixture. Thereafter, 110 pots were inoculated with 12 g vital *F. mosseae* inoculum (Mycorrhizal plants, M) that had been mixed with 0.5 g sterilized bonemeal (16 % phosphate, Ecostyle, The Netherlands) and 7.5 g fully mixed sterile soil-sand-sorbix mixture. Bonemeal was used as a slow-release source of phosphorus in the experiment to promote mycorrhizal performance. Its addition resulted in 133 mg of total P per kg of soil, corresponding to ca. 4 mg of water-soluble P per kg of soil (Ylivainio et al. 2008). The other 96 pots were inoculated with 12 g autoclaved (30 min at 121 °C) *F. mosseae* inoculum mixed with 0.5 g sterilized bonemeal (Ecostyle, The Netherlands) and 7.5 g sterile soil-sand-sorbix mixture (Non-mycorrhizal plants, NM). One seedling then was planted into each pot, and pots were watered three times per week (two times using demineralized water and one time using 50 ml of a 0.5 strength Hoagland solution without phosphate). Five weeks after transplantation, the number of main rosette leaves on each plant was counted, and the length of each leaf was measured. Plant size was determined by calculating the total leaf length of each plant to enable an equal initial plant size distribution for each treatment when allocating plants to the different timing and herbivore treatments within the mycorrhizal and non-mycorrhizal groups (see below).

The experiment was set up to examine the effects of mycorrhizal infection, aboveground herbivory, and the timing of aboveground herbivory on the performance of a later feeding aboveground herbivore and on induced plant defense. To standardize the amount of damage caused by the ‘treatment’ herbivores, two clip-cages (2 cm diam), each with one fourth instar *S. exigua* were simultaneously placed on the distal part of the seventh youngest fully expanded mature leaf for 24 h. During this time, two areas of 3.14 cm^2^ were consumed. The herbivory treatment was initiated at five different times: 8, 4, 2, and 1 d before the introduction of response herbivore (see below). The 8-d-treatment was initiated 5 wk. after transplantation. Empty clip-cages were put on subsets of the (no-herbivory) control plants at 8, 4, 2, and 1 d before introduction of response caterpillars. The experiment followed a full factorial design with 2 AMF treatments (M = mycorrhizal, NM = non-mycorrizal) and 5 herbivory treatments (8 d, 4 d, 2 d, 1 d, control = no treatment herbivory). Of the 206 plants in total, 135 plants (15 replicates for herbivory treatments and 12 replicates for control within M plants; 13 replicates for herbivory treatments and 11 replicates for controls within NM plants) were used to examine the effects of mycorrhizal presence, herbivory, and timing of herbivory on subsequent herbivore performance and induced plant defense. Effects on subsequent herbivores were tested in two bioassays described below. The 71 remaining plants (8 M and 7 NM replicates for each of the four herbivory treatments, and 6 M and 5 NM replicates for their respective controls) were not subjected to any bioassay but used to assess plant biomass production as a function of AMF and induction by treatment caterpillars.

### Bioassays

The effects of AMF association, previous herbivory, and timing of previous herbivory on later arriving herbivores were examined using two bioassays.

### Detached Leaf Bioassay

For this bioassay, two leaves (the fifth and sixth youngest true leaf) of each of the 135 bioassay plants (leaf number: mean = 9.94; SE = 0.07) were excised and weighed at *t* = 0 (8, 4, 2, and 1 d after the respective 24 h herbivory treatments, 43 d after seedling transplantation). From each leaf, 3 leaf disks were taken around the mid-vein using a sharp cork borer (16 mm diam) so that a total of 6 leaf cuttings were obtained from each plant (Biere et al. 2004). Two of these six leaf disks (one from each leaf) were used to determine fresh weight and dry weight (after drying for 72 h at 50 °C). The four remaining disks were placed on moist filter paper in a Petri dish (9 cm diam) where a freshly-moulted pre-weighed 3rd instar *S. exigua* (“bioassay” or response caterpillar, mean = 9.66 mg; SE = 0.07) was introduced. The Petri dishes were placed in a growth chamber at 25 °C and a photoperiod of 16/8 h (L/D). After exactly 24 h, the larvae were removed and immediately reweighed and then frozen (−20 °C). Frozen caterpillars were oven-dried at 50 °C, and their dry weight was determined. The remaining, non-consumed material of the leaf disks was collected and scanned using a photo scanner (EPSON, PERFECTION 4990, Japan) to determine the leaf area consumed by the bioassay caterpillar using the software WinFOLIA (Regent Instruments, Sainte-Foy, Canada). The remaining leaf disks were oven-dried at 50 °C to enable estimation of the leaf dry weight consumed by bioassay caterpillars. The remaining plant material of the two leaves from which leaf disks were taken was oven-dried (50 °C) and used for chemical analysis (see below). Detached leaf 24-h bioassays have been successfully applied in this system before (Biere et al. 2004) and have shown good correlations between IG concentrations of leaves at the time of detachment and *S. exigua* performance on detached leaves. This indicates that even though absolute levels of primary or secondary metabolites may differ between attached and detached leaves, the latter are still likely to represent relevant differences in leaf chemical quality between the plants in the experiment.

### Whole Plant Bioassay

After excision of two leaves from the 135 plants used for the detached leaf bioassay at day 0, these plants were individually caged using cylindrical mesh cages (height 1 m, diam 35 cm) for the second bioassay, assessing their responses to previous herbivory in the longer term (8 d extra, see below). One pre-weighed 3rd instar *S. exigua* (mean = 24.0 mg; SE = 0.32) was introduced into each of the 135 cages with bioassay plants. The larvae could move freely within the cage. Eight days later, the surviving caterpillars were collected, reweighed, and oven-dried. Mortality was high in the cages, and dead larvae also were collected and oven-dried. After collection of the caterpillars, the 135 bioassay plants were harvested. All leaves of each plant were scanned, and the leaf area consumed by the response caterpillars was determined using the same equipment and software as described above for the detached leaf bioassay. Roots were removed carefully from the soil and rinsed. A small sample of the roots was taken from nine randomly selected mycorrhizal and five non-mycorrhizal plants that had not been subjected to previous herbivory to quantify the extent of root colonization by *F. mosseae*. Leaf and root material was oven-dried (50 °C), and dry weight was determined. The 71 plants not used in the bioassays were caged as well and harvested simultaneously with the bioassay plants.

### Iridoid Glycoside Analysis

All 135 leaf samples of the bioassay plants were weighed and ground. Twenty-five mg of each sample were extracted overnight in 70 % methanol, and then filtered (12–15 μm) followed by a dilution of 10 times with ultrapure water. The concentrations of the IGs aucubin and catalpol were analyzed using HPLC as described by Marak et al. (2002).

### Caterpillar Performance

For the detached-leaf bioassay, three indices were calculated to characterize herbivore performance following Waldbauer (1968). Relative growth rate of caterpillars was calculated as RGR = (CDW2-CDW1)/(0.5*(CDW1 + CDW2)), where CDW1 and CDW2 are initial and final (after 24 h) dry weight of caterpillars and CDW1 of each caterpillar was estimated from its initial fresh weight and its final fresh:dry weight ratio. The relative consumption rate of caterpillars was calculated as RCR = (LDW2-LDW1)/(0.5*(CDW1 + CDW2)), where LDW1 and LDW2 are initial and final dry weight of the four leaf disks, and LDW1 for each plant was calculated from the initial fresh weight of the four leaf disks and the initial fresh:dry weight ratio of the two leaf disks from the corresponding plant. The efficiency of conversion of ingested food (ECI) was calculated as (CDW2-CDW1)/(LDW2-LDW1).

### Plant Size and Biomass

Number of rosette leaves and maximum leaf length of all 206 plants were used to analyze effects of AMF on plant size 5 wk. after transplantation, prior to herbivore treatments. Plant biomass of the 71 plants not used in the bioassays was used to analyse effects of AMF and induction by 24 h of feeding by treatment caterpillars on plant dry weight production in the absence of caterpillar feeding 7 wk. after transplantation. Leaf mass was corrected for the dry weight of the two leaf discs that were removed for the early herbivory treatment, estimated based on the area:dry weight ratio of the excised leaf discs for the bioassay of the corresponding plant. Leaf and root biomass of the 135 bioassay plants was used to analyze the effects of AMF and induction by treatment caterpillars on plant dry weight production in the presence of caterpillars feeding for an 8 d period. Leaf dry weight of these plants was corrected by adding the dry weight of the two excised leaves based on the fresh weight:dry weight ratio of the remainder of these leaves after removing the six leaf disks. Root dry weights of the plants from which subsamples were used for examining mycorrhizae were corrected by adding the dry weight of these subsamples (estimated on the basis of the the root fresh:dry weight ratio for the corresponding plants) to the root dry weights of these plants.

### Root Colonization by AMF

Colonization of roots by *F. mosseae* was quantified using the gridline intersect method (McGonigle et al. 1990). Briefly, at least 100 small root pieces per root sample were cleared in 10 % KOH for 10 min at 95 °C, and stained with a mixture of vinegar (5 % acetic acid) and 5 % Scheaffer black ink for 8 min at 80–90 °C. Stained roots were mounted on slides and checked for confirmation of mycorrhizal colonization of plants in the mycorrhizal treatment and absence of mycorrhizal colonization in control plants (Vierheilig et al. 1998) under a compound microscope (BH-2; Olympus, Tokyo, Japan) at ×40 magnification. The presence of AMF structures (hyphae, arbuscules, vesicles, or spores) was scored at 120 grid intersections per root sample, and the scores were averaged per plant.

### Statistical Analysis

To determine how AMF, previous herbivory (the 24 h period of feeding by treatment caterpillars) and the timing of previous herbivory affected the performance of response (bioassay) caterpillars and leaf IG concentrations in the detached leaf bioassay, we performed three-way *ANOVA*s in which AMF status (presence or absence of mycorrhizal fungi) and previous herbivory (presence or absence of treatment herbivory) were used as categorical factors and the timing of previous herbivory (or an empty clip cage), 8, 4, 2, or 1 d before introduction of bioassay caterpillars, was included as a continuous variable. Due to high mortality of caterpillars in the whole plant bioassay, possibly partly caused by pathogen infestation, no attempts were made to analyze caterpillar performance for this bioassay. Instead, differences in survival between AMF and non-AMF plants were analyzed using generalized linear models with a binomial distribution and logit link function.

To determine the effects of AMF and induction by a 24 h period of caterpillar feeding (previous herbivory) on shoot and root biomass of the 71 plants that were not used in the bioassays, we used a two-way *ANOVA* with AMF (presence or absence) and previous herbivory (herbivory at 8, 4, 2, or 1d before introduction of bioassay caterpillars and no herbivory) as fixed factors. The data did not allow a full three-way analysis with AMF, previous herbivory, and timing of previous herbivory, since there were only one or two replicates for the no-herbivory control (empty clip-cage) treatment per time point for these 71 plants. Instead, the replicates within the no-herbivory treatment for each time point were grouped together as one level (“no previous herbivory”) of the factor previous herbivory. Note that effects of previous herbivory in this analysis are indicative of costs of induction rather than costs of leaf removal since the leaf area that was removed by treatment caterpillars from induced plants was added to the leaf biomass. Similarly we tested effects of AMF and previous herbivory (8, 4, 2, or 1d before the introduction of bioassay caterpillars and no herbivory) on root and shoot biomass of the 135 plants that were used in the bioassays. For all data, the residuals were checked for normality using a Kolmogorov-Smirnov one-sample test, and for homogeneity of variance using a Levene test before analysis and transformed when necessary.

## Results

### Effects of AMF and Previous Herbivory on Plant Biomass

**Fig. 1:**
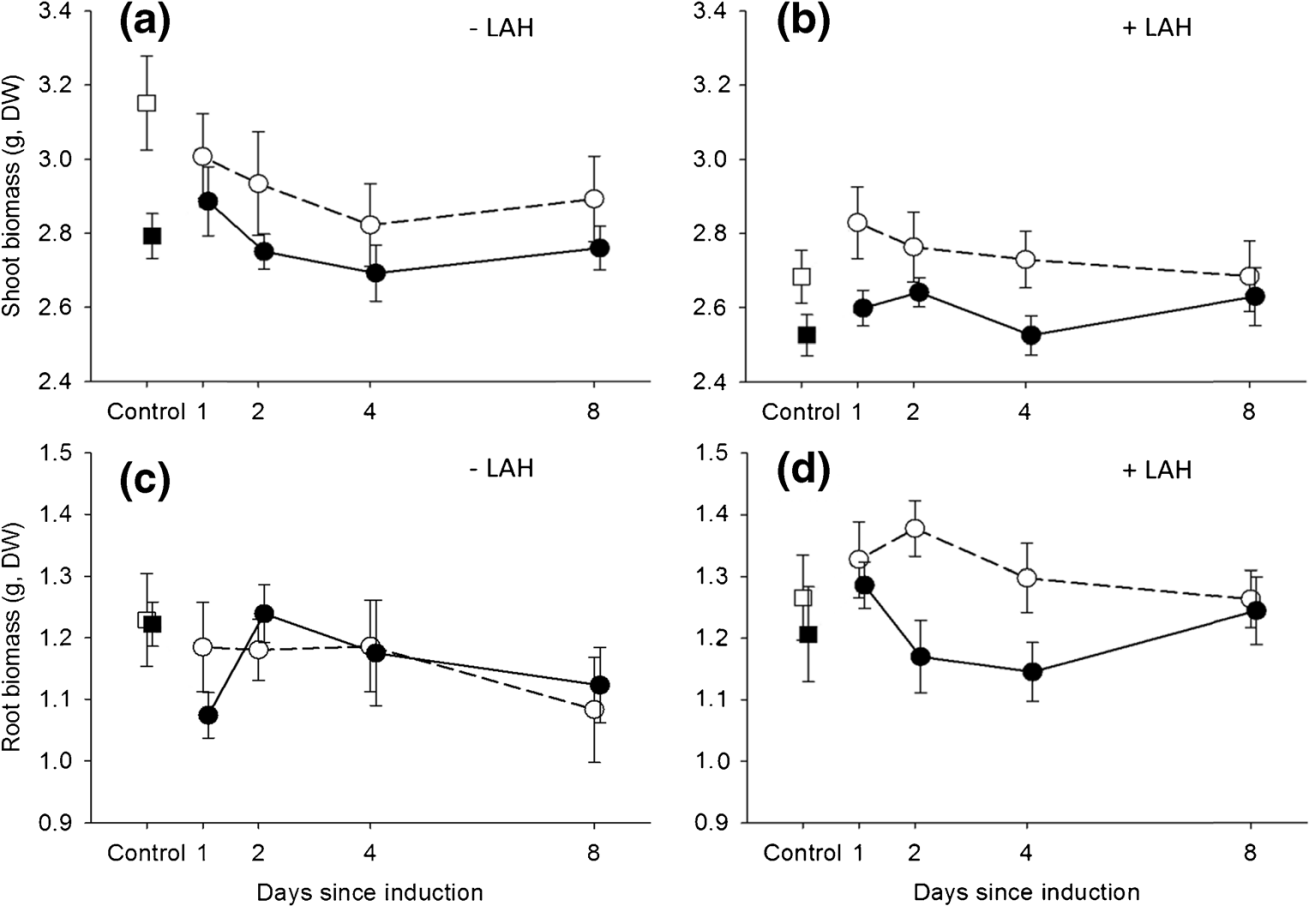
Mean (±SE) shoot (**a, b**) and root (**c, d**) biomass of mycorrhizal (*filled symbols*) and non-mycorrhizal (*open symbols*) *Plantago lanceolata* plants that were (*circles*) or were not (*squares*) exposed to previous herbivory 1, 2, 4, and 8 d prior to the introduction of later arriving herbivores (LAH) and that were (+LAH) or were not (−LAH) exposed to an eight-day period of feeding by later arriving herbivores prior to harvest. Filled symbols: *N* = 15 for +LAH and *N* = 8 for −LAH; open symbols: *N* = 13 for +LAH and *N* = 7 for −LAH. See Table 1 for statistics. Note: the controlled amount of leaf biomass removed by the previous treatment herbivores has been added to the shoot biomass

Arbuscular mycorrhizal fungi did not affect plant size, measured as total leaf length, at the age of five weeks, just prior to the herbivory treatments (*F*_*1*,*204*_ = 2.16, *P* = 0.144). However, AMF had minor effects on plant morphology. Specifically, AMF plants produced a slightly larger number of main rosette leaves (10.2 vs. 9.9, *F*_*1*,*204*_ = 5.96, *P* = 0.015) at the expense of a slightly smaller maximum leaf length (20.0 vs. 20.6 cm, *F*_*1*,*204*_ = 4.60, *P* = 0.033). However, at the age of 7 weeks, AMF had significantly reduced the shoot biomass of the plants, both the ones that had not been used for the bioassays (by on average 7.1 %, Table 1, *P* < 0.001, Fig. 1a) and the ones that had been used for the bioassays (by on average 6.8 %, Table 1, *P* < 0.001; Fig. 1b). Induction of plants by treatment caterpillars did not significantly affect the shoot biomass of these plants (Table 1, Fig. 1a). Root biomass was not affected by either AMF or previous herbivory (Table 1, Fig. 1c). A similar pattern was observed for the plants that had been exposed to an eight-day period of feeding by later arriving herbivores except that AMF also had a negative effect on root biomass. On these plants, AMF reduced the shoot and root biomass by on average 6.8 % (Table 1, *P* < 0.001, Fig. 1b) and 7.4 %, respectively (Table 1, Fig. 1d). There were no effects of induction of plants by previous herbivory on shoot or root biomass, nor any interactions between AMF and previous herbivory (Table 1). Similar results were obtained when we specifically tested the contrast between “no herbivory” (empty clip cage plants) and “previous herbivory” (all other levels of this factor, i.e., previous herbivory at 1, 2, 4, and 8 d before introduction of bioassay caterpillars combined) and its interaction with AMF (all *P* > 0.09).

**Table 1:** ANOVA results for impacts of arbuscular mycorrhizal fungi (AMF) inoculation and previous herbivory on the shoot and root biomass of *Plantago lanceolata* in the absence or presence of an eight-day feeding period by later arriving herbivores (LAH)

		Non-bioassay (−LAH)					Bioassay (+LAH)			
		Shoot mass		Root mass			Shoot mass		Root mass	
	*df1*	*F*	*P*	*F*	*P*	*df2*	*F*	*P*	*F*	*P*
AMF (M)^a^	1	**11.06** ^c^	**0.001** ^c^	0.02	0.884	1	**23.37** ^c^	**<0.001** ^c^	**7.42** ^c^	**0.007** ^c^
Herbivory^b^ (H)	4	0.82	0.520	1.24	0.303	4	1.29	0.276	0.74	0.570
M*H	4	0.77	0.550	0.54	0.710	4	0.82	0.512	1.08	0.369
Error	61					125				

^a^AMF inoculation indicates two treatment groups with vital or autoclaved sterile AMF

^b^Herbivory refers to treatments within non-AMF or AMF groups that were exposed to previous herbivory 1, 2, 4 and 8 d prior to the introduction of bioassay caterpillars or no herbivory

^c^Bold values indicate significant effects at *P* < 0.05

### Effect of AMF, Previous Herbivory, and Timing of Previous Herbivory on Caterpillar Performance

Roots of plants from the mycorrhizal treatment that had not experienced herbivory showed low but consistent levels of colonization by AMF structures (hyphae, arbuscules, and vesicles, 22.9 ± 2.5 %, *N* = 9). In the control treatment, no *F. mosseae* structures were found (*N* = 5).

### Detached-Leaf Bioassay

**Fig. 2:**
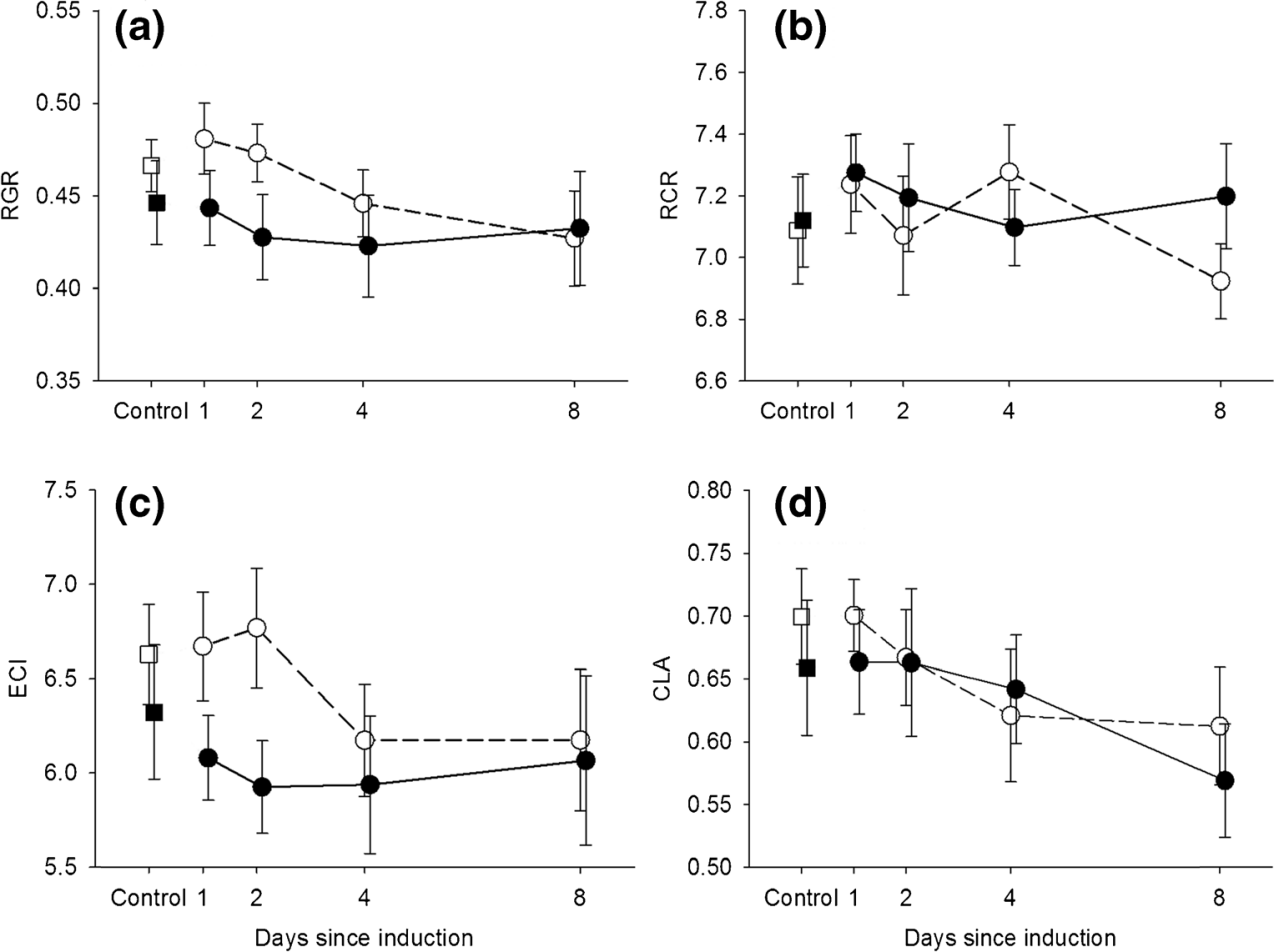
Mean (± SE) relative growth rate (RGR, **a**), relative consumption rate (RCR, **b**), efficiency of conversion of ingested food (ECI, **c**), and consumed leaf area (CLA, **d**) of bioassay caterpillars after 24 h of feeding on excised leaves of mycorrhizal (*filled symbols*, *N* = 15) and non-mycorrhizal (*open symbols*, *N* = 13) *Plantago lanceolata* plants. Plants had either been exposed to no herbivory (Control, *squares*), or to a controlled 24 h period of herbivory 1, 2, 4, or 8 d prior to the bioassay (*circles*). See Table 2 for statistics

AMF colonization significantly reduced the relative growth rate (RGR) of bioassay caterpillars in the detached-leaf bioassay (*F*_*1*,*127*_ = 4.45, *P* = 0.037, Table 2, Fig. 2a). Differences in RGR among caterpillars could be explained mainly by variation in the efficiency with which they converted the ingested food into biomass (ECI, explaining 85.3 % of variation), whereas variation in their relative consumption rates (RCR) explained very little variation in RGR (1.5 %). Although this suggests that AMF reduced food quality rather than intake rates, effects of AMF on neither of these two individual components of RGR were statistically significant (Table 2, Fig. 2b, c). In accordance with the negligible contribution of RCR to differences in RGR, the negative effect of AMF on caterpillar RGR was not reflected in a reduced rate of leaf area consumption (Table 2, Fig. 2d). Previous herbivory did not have a significant main effect on leaf area consumption by bioassay caterpillars (Table 2). However, interestingly, the effect of previous herbivory on leaf area consumption significantly increased over time (Table 2, *P* = 0.04), from no reduction observed when herbivory occurred one day earlier, to 13 and 14 % reduction in leaf area consumption when herbivory occurred eight days earlier, for non-mycorrhizal and mycorrhizal plants, respectively. AMF did not interact with the plant’s response to previous herbivory (no AMF × herbivory, nor AMF × herbivory × time interactions, Table 2) in terms of leaf area consumption by bioassay caterpillars or their RGR.

**Table 2:** ANOVA results for effects of AMF inoculation, previous herbivory and timing of induction on relative growth rates (RGR), relative consumption rates (RCR), efficiency of conversion of ingested food (ECI) and consumed leaf area (CLA) of bioassay caterpillars

		RGR		RCR		ECI		CLA (cm^2^)	
	*df*	*F*	*P*	*F*	*P*	*F*	*P*	*F*	*P*
AMF (M)^a^	1	**4.45** ^d^	**0.037** ^d^	1.32	0.253	2.69	0.104	0.27	0.062
Herbivory (H)^b^	1	1.10	0.295	0.46	0.498	0.47	0.496	0.73	0.396
Time (T)^c^	1	0.06	0.815	0.00	0.960	0.05	0.816	**4.32** ^d^	**0.040** ^d^
M*H	1	0.32	0.574	0.93	0.337	0.00	0.992	0.39	0.534
M*T	1	2.37	0.127	3.77	0.054	0.49	0.487	0.99	0.321
H*T	1	1.91	0.170	0.83	0.365	0.91	0.342	0.13	0.717
M*H*T	1	0.38	0.541	1.94	0.167	0.02	0.890	0.69	0.407
Error	127								

^a^Arbuscular mycorrhizal fungi (AMF) inoculation indicates two treatment groups with vital or autoclaved sterile AMF

^b^Herbivory refers to plants that were or were not exposed to a 24 h period of herbivory prior to introduction of bioassay caterpillars

^c^Time refers to when plants assigned to previous herbivory treatments were exposed to herbivory or empty clip cages (8, 4, 2, and 1 d before the introduction of bioassay caterpillars)

^d^Bold values indicate significant effects at *P* < 0.05

### Whole-Plant Bioassay

Bioassay caterpillars in the whole-plant bioassay (that fed on caged plants for eight days) suffered unexpectedly high levels of mortality (57.8 %), which precluded further analysis of effects of AMF and previous herbivory on their performance. Survival rates were significantly higher on mycorrhizal plants (50.0 %) than on non-mycorrhizal plants (33.3 %) (*Wald* = 7.93, *P* < 0.005), both on plants that had experienced previous herbivory (60.0 vs. 32.7 %) and plants that had not (75.0 vs. 36.4 %). There was no significant effect of previous herbivory on survival (*Wald* = 2.41, *P* = 0.12), nor an interaction between AMF and previous herbivory (*Wald* = 3.01, *P* = 0.08).

### Effects of AMF, Previous Herbivory, and Timing of Previous Herbivory on Shoot IG Concentration

**Fig. 3:**
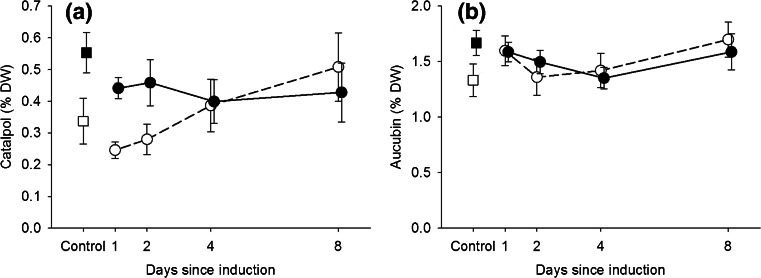
Mean (±SE) shoot catalpol (**a**) and aucubin (**b**) concentration of mycorrhizal (*filled symbols*, *N* = 15) and non-mycorrhizal (*open symbols*, *N* = 13) *Plantago lanceolata* plants that had experienced no herbivory (Control, *squares*) or a controlled 24 h period of herbivory 1, 2, 4, or 8 d prior to the bioassay (*circles*). See Table 3 for statistics

Overall, AMF colonization of plant roots increased the shoot concentration of catalpol (*F*_*1*,*127*_ = 7.17, *P* = 0.008, Fig. 3a, Table 3), whereas the increase in the shoot concentration of aucubin was not significant (*F*_*1*,*127*_ = 3.43, *P* = 0.066, Fig. 3b, Table 3). Neither herbivory nor the timing of herbivory had a significant effect on shoot concentrations of aucubin or catalpol (Table 3). However, when we specifically focus on the plants that had been subjected to previous herbivory, an interesting pattern arises. In non-mycorrhizal plants, the concentration of catalpol significantly increased ca. two-fold over the time period between one and eight days following exposure to herbivory (linear regression, *F*_*1*,*50*_ = 8.02, *P* = 0.007). By contrast, mycorrhizal plants, that had already 64 % higher leaf catalpol concentrations at the start of the herbivory treatment (Fig. 3b, squares, *F*_*1*,*26*_ = 5.10, *P* = 0.033), did not show a further increase following herbivory (linear regression, *F*_*1*,*58*_ = 0.07, *P* = 0.796), resulting in a significant interaction between presence or absence of AMF and timing of previous herbivory for plants exposed to herbivory (*F*_*1*,*108*_ = 5.56, *P* = 0.020). No such effects were observed for aucubin.

**Table 3:** ANOVA results for impacts of arbuscular mycorrhizal fungi (AMF) inoculation, previous herbivory and timing of induction on the concentration of aucubin and catalpol in leaves of *Plantago lanceolata* in the absence of later herbivore feeding

		Aucubin		Catalpol	
	*df*	*F*	*P*	*F*	*P*
AMF (M)^a^	1	3.43	0.066	**7.17** ^d^	**0.008** ^d^
Herbivory (H)^b^	1	0.15	0.699	0.15	0.695
Time (T)^c^	1	0.66	0.419	2.49	0.117
M * H	1	2.10	0.150	0.18	0.672
M* T	1	1.06	0.305	1.79	0.183
H * T	1	0.01	0.925	0.01	0.926
M * H * T	1	0.22	0.637	0.19	0.665
Error	127				

^a^AMF inoculation indicates two treatment groups with vital or autoclaved sterile AMF

^b^Herbivory refers to plants that were or were not exposed to a 24 h period of herbivory prior to introduction of bioassay caterpillars

^c^Time refers to when plants assigned to previous herbivory treatments were exposed to herbivory or empty clip cages (8, 4, 2, and 1 d before the introduction of bioassay caterpillars)

^d^Bold values indicate significant effects at *P* < 0.05

## Discussion

Our study of interactions between the host plant *P. lanceolata*, the arbuscular mycorrhizal fungus *F. mosseae*, and the foliar insect herbivore *S. exigua* showed that root colonization by the fungus (1) reduces the shoot biomass of the host plant, (2) systemically induces a defense metabolite (catalpol) in the shoots of the host plant, (3) alters the time course of induction of this defense metabolite by the host plant in response to foliar insect herbivory, and (4) reduces the relative growth rate of later arriving conspecific foliar insect herbivores.

### Effects of AMF and Shoot Herbivory on Plant Biomass

In contrast to our hypotheses, induction of plants by *S. exigua* did not reduce shoot biomass, neither in the presence, nor in the absence of AMF. Since similar, very small, amounts of leaf tissue were removed from plants exposed to the 24-h period of previous herbivory and from control plants, the absence of effects of previous herbivory on shoot biomass indicates that there were no costs of induction, rather than no costs of leaf removal. Such costs may be small under the no-competition and relatively high resource conditions as in our experiment (Cipollini et al. 2003). In our study, shoot biomass of mycorrhizal plants was lower than that of non-mycorrhizal plants, independent of whether the plants were exposed to previous herbivory or not (no interaction between previous herbivory and AMF). It has long been recognized that AMF can not only have positive effects on host plant growth, but can also negatively affect plant growth under a large set of environmental conditions (Johnson et al. 1997; Klironomos 2003). Our results corroborate previous studies in *P. lanceolata* showing a continuum of mycorrhizal growth responses (the difference in biomass between mycorrhizal and non-mycorrhizal plants weighted by that of non-mycorrhizal plants) from positive or neutral (e.g., Karasawa et al. 2012; Zaller et al. 2011) to negative (Ayres et al. 2006). Negative growth responses can result from costs for the plant of maintaining the symbiosis that exceed the benefits, particularly under conditions of high soil nutrient availability, low light intensity, or weak mutual coadaptation (Johnson et al. 1997; Klironomos 2003). Lower shoot biomass may result from a mycorrhiza-induced reallocation of photosynthates from shoot to root tissue due to the higher demands of resources in roots for maintaining the mycorrhizal association. However, mycorrhizal plants in our study did not possess higher root mass either. Instead, root mass was even lower when plants were subsequently exposed to later arriving herbivores (Fig. 1d). This may indicate that higher levels of shoot consumption by later arriving herbivores further limited plant photosynthesis and thereby restricted photosynthate allocation to root tissues in mycorrhizal plants already constrained in carbon by mycorrhizal colonization. Alternatively, herbivory may have maintained plants in an induced state, conserving resources in shoots for induced defense instead of roots where mycorrhizae may directly compete for these resources.

### AMF Colonization Influences Plant IG Induction and Response Herbivore Performance

Several studies have indicated that AMF can enhance resistance against particular groups of foliar feeding herbivores, a phenomenon known as Mycorrhiza-Induced Resistance (MIR, Jung et al. 2012; Pozo and Azcón-Aguilar 2007). MIR is observed mainly for generalist, chewing insect herbivores (see reviews by e.g., Cameron et al. 2013; Jung et al. 2012; Koricheva et al. 2009). MIR can result from changes in primary metabolites as well as from systemic induction or jasmonic acid-dependent priming of defense metabolites (e.g., Garcia-Garrido and Ocampo 2002; Jung et al. 2012; Song et al. 2013). In *P. lanceolata*, variable effects of AMF have been observed regarding the systemic induction of its main defense metabolites, iridoid glycosides, and the ability of plants to induce these defenses in response to later arriving herbivores (Bennett et al. 2009; Fontana et al. 2009; Gange and West 1994; Schweiger et al. 2014). Our results resemble those of Bennett et al. (2009) obtained for the AMF *Scutellospora calospora* that systemically induced iridoid glycosides in the leaves of *P. lanceolata*, but suppressed a further induction of these compounds in response to herbivory. Other studies have reported either no systemic induction or even a decrease in IGs in AMF-colonized plants (Bennett et al. 2009; Fontana et al. 2009; Schweiger et al. 2014). In our study, it was mainly the concentration of catalpol that was induced by AMF, the more toxic of the two iridoid glycosides present in *P. lanceolata*, indicating that AMF can cause shifts in both the levels and in the relative proportions of iridoid glycosides in *P. lanceolata* (cf*.* Bennett et al. 2013). AMF caused a significant reduction in the relative growth rate (RGR) of later arriving caterpillars, which may or may not have been mediated by the induced changes in the levels of catalpol. The reduction in RGR is consistent with the occurrence of MIR against generalist chewing foliar herbivores such as *S. exigua*. Interestingly, the AMF-induced reduction in caterpillar RGR was not accompanied by a lower relative consumption rate (RCR), and there was no significant effect of AMF on leaf area consumption. This indicates that at least in the short-term, the negative effect of AMF on *S. exigua* may have been mediated by a lower leaf quality rather than a lower feeding rate. The plant’s association with AMF may therefore not directly benefit the plant in terms of reduced feeding rates of the caterpillars. However, it may potentially incur benefits in the longer run if the reduction in RGR results in lower rates of herbivore development and population growth.

### AMF Modulate the Magnitude and Timing of the Defense Response of Plants to Herbivory

Previous herbivory resulted in a reduction in leaf area consumption by later arriving herbivores when sufficient time had passed since induction took place, i.e., the effect increased over the eight-day period since the short term exposure to inducing herbivores. This indicates that herbivory results in the gradual induction of defenses that affects the consumption rate by later arriving herbivores. One of the most interesting findings of our study is that the induction of defense metabolites in response to the 24 h period of herbivory strongly differed between mycorrhizal and non-mycorrhizal plants. When considering the subset of plants that had been exposed to previous herbivory, non-mycorrhizal plants showed a linear increase in their leaf levels of catalpol over the eight-day period, whereas mycorrhizal plants did not. One way to interpret these results is that mycorrhizae, instead of priming *P. lanceolata* plants for herbivore-induced biosynthesis of defense chemicals, actually repressed the induction of these metabolites by herbivores. Mycorrhizal suppression of the ability of plants to induce defense chemicals has been observed in *P. lanceolata* both with respect to the induction of volatile organic compounds (VOC) potentially involved in indirect defense (Fontana et al. 2009), and with respect to the induction of iridoid glycosides (Bennett et al. 2009). The extent and direction of the modulation of defense responses to herbivory in *P. lanceolata* is AMF species dependent (Bennett et al. 2009) and further study is necessary to elucidate what governs the continuum from AMF-dependent priming to AMF-dependent repression of herbivore-induced responses in plants.

An alternative explanation for the observed lack of an herbivore-induced increase in catalpol in mycorrhizal plants in our experiments could be that in mycorrhizal plants the systemic induction of catalpol (prior to herbivory) had already resulted in the maximum amount of catalpol that could be attained in the foliage under the prevailing conditions. However, given the overall low levels of catalpol compared to levels observed in other experiments (e.g., Bennett et al. 2009; De Deyn et al. 2009), this does not seem to be a very likely explanation.

As a result of the failure of mycorrhizal plants to induce catalpol in response to herbivory, the initial difference in leaf catalpol concentrations between mycorrhizal and non-mycorrhizal plants (that did induce this compound in response to herbivory) completely disappeared after four days following herbivory. This pattern corresponds well with the observed time course of RGR and ECI of response caterpillars that initially tended to be higher on non-mycorrhizal than on mycorrhizal plants, but dropped to levels that were as low as on the mycorrhizal plants (Fig. 2a, c) since four days after the herbivory. Although it is tempting to speculate that there is a causal connection between the time course of the increase in catalpol and decrease in ECI and RGR, it should be noted that the later time trend was not statistically significant. Moreover, the design of our study only allows us to speculate about the role of catalpol in mediating effects of AMF on caterpillar performance; any causal relationship is awaiting further study. Artificial diet studies have provided strong evidence that catalpol can reduce the relative growth rate of caterpillars of generalist insect herbivores including *Spodoptera* species (Bowers and Puttick 1988; Puttick and Bowers 1988). However, AM fungi are known to cause strong metabolic reprogramming of shoots; recent studies in *P. lanceolata* have shown that more than 5 % of identified metabolic features changes in response to root colonization by the AM fungus *Rhizophagus irregularis* (Schweiger et al. 2014). Therefore, there are probably many potential primary or secondary metabolites that could contribute to the AMF effects on herbivore growth rates. Furthermore, IGs represent a dual defense system. Upon damage, these compounds are activated by their specific beta-glucosidases (Pankoke et al. 2013). Currently, it is unknown whether the activity of these beta-glucosidases is affected by AMF and/or herbivory. If this is the case, however, understanding the role of IGs in mediating such interactions may be rather complex.

In summary, mycorrhizal plants had higher catalpol levels when herbivores arrived, while non-mycorrhizal plants only gradually built up this defense. Interestingly, this pattern was not explained by AMF priming of defense, but by the combination of two different AMF effects, i.e., early systemic induction and subsequent repression of the plant’s ability to exhibit an herbivore-induced response. Its causal role in modulating the herbivore response awaits further study.

In summary, AMF caused a reduction in plant biomass, but also resulted in a systemic increase in the concentration of defense metabolites in the shoots of *P. lanceolata.* This may have contributed to the negative impact of AMF on the performance of later-arriving shoot herbivores. Non-mycorrhizal plants only reached these levels of defense metabolites eight days after induction by herbivores, while levels of defense compounds in mycorrhizal plants were not affected by herbivory. Our study thus reveals that AMF can modulate the time course of effects of previous herbivory on plant responses to, and performance of, later arriving herbivores, which may in turn determine plant performance and fitness in the longer run. This highlights the importance of including temporal aspects in future research on interactive aboveground-belowground impacts of herbivory and AMF on expression and effects of induced plant defenses.
